# Alpha-1 antitrypsin Pi∗Z allele is an independent risk factor for liver transplantation and death in patients with advanced chronic liver disease

**DOI:** 10.1016/j.jhepr.2022.100562

**Published:** 2022-08-20

**Authors:** Lorenz Balcar, Bernhard Scheiner, Markus Urheu, Patrick Weinberger, Rafael Paternostro, Benedikt Simbrunner, Lukas Hartl, Mathias Jachs, David Bauer, Georg Semmler, Claudia Willheim, Matthias Pinter, Peter Ferenci, Michael Trauner, Thomas Reiberger, Albert Friedrich Stättermayer, Mattias Mandorfer

**Affiliations:** 1 Division of Gastroenterology and Hepatology, Department of Internal Medicine III, Medical University of Vienna, Vienna, Austria; 2 Vienna Hepatic Hemodynamic Lab, Medical University of Vienna, Vienna, Austria

**Keywords:** AATD, cirrhosis, prognostication, genetic risk, rare disease, AAT, Alpha-1 antitrypsin, AATD, Alpha-1 antitrypsin deficiency, ACLD, Advanced chronic liver disease, CTP, Child-turcotte-pugh score, ER, Endoplasmic reticulum, GWAS, Genome wide association studies, HCC, Hepatocellular carcinoma, (a[S])HR, (Adjusted [subdistribution]) hazard ratio, HVPG, Hepatic venous pressure gradient, NAFLD, Non-alcoholic fatty liver disease, SERPINA1, Serpin family a member 1, UNOS MELD (2016), United network for organ sharing model for end-stage liver disease

## Abstract

**Background & Aims:**

Alpha-1 antitrypsin (AAT) deficiency causes/predisposes individuals to advanced chronic liver disease (ACLD). However, the role of the *SERPINA1* Pi∗Z allele in patients who have already progressed to ACLD is unclear. Thus, we aimed to evaluate the impact of the Pi∗Z allele on the risk of liver transplantation/liver-related death in patients with ACLD, while adjusting for the severity of liver disease at inclusion.

**Methods:**

A total of 1,118 patients with ACLD who underwent hepatic venous pressure gradient (HVPG) measurement and genotyping for the Pi∗Z/Pi∗S allele at the Vienna Hepatic Hemodynamic Lab were included in this retrospective analysis. The outcome of interest was liver transplantation/liver-related death, while non-liver-related death and removal/suppression of the primary etiological factor were considered as competing risks.

**Results:**

Viral hepatitis was the most common etiology (44%), followed by alcohol-related (31%) and non-alcoholic fatty liver disease (11%). Forty-two (4%) and forty-six (4%) patients harboured the Pi∗Z and Pi∗S variants, respectively. Pi∗Z carriers had more severe portal hypertension (HVPG: 19±6 *vs.*15±7 mmHg; *p <*0.001) and hepatic dysfunction (Child-Turcotte-Pugh: 7.1±1.9 *vs.* 6.5±1.9 points; *p =* 0.050) at inclusion, compared to non-carriers. Contrarily, the Pi∗S allele was unrelated to liver disease severity. In competing risk regression analysis, harbouring the Pi∗Z allele was significantly associated with an increased probability of liver transplantation/liver-related death, even after adjusting for liver disease severity at inclusion. The detrimental impact of the common Pi∗MZ genotype (adjusted subdistribution hazard ratio: ≈1.56 *vs.* Pi∗MM) was confirmed in a fully adjusted subgroup analysis. In contrast, Pi∗S carriers had no increased risk of events.

**Conclusion:**

Genotyping for the Pi∗Z allele identifies patients with ACLD at increased risk of adverse liver-related outcomes, thereby improving prognostication. Therapies targeting the accumulation of abnormal AAT should be evaluated as disease-modifying treatments in Pi∗Z allele carriers with ACLD.

**Lay summary:**

Alpha-1 antitrypsin deficiency is a genetic disease that affects the lung and the liver. Carrying two dysfunctional copies of the gene causes advanced liver disease. Harbouring one dysfunctional copy increases disease severity in patients with other liver illness. However, the significance of this genetic defect in patients who already suffer from advanced liver disease is unclear. Our study found that harbouring at least one dysfunctional copy of the alpha-1 antitrypsin gene increases the risk of requiring a liver transplantation or dying from a liver disease. This indicates the need for medical therapies aimed at treating the hepatic consequences of this genetic defect.

## Highlights

• Pi∗Z allele is significantly associated with liver-related events in patients with ACLD.
• This finding extends to patients harbouring the Pi∗MZ genotype.
• Genotyping for the Pi∗Z allele might improve prognostication in patients with ACLD.
• Therapies targeting accumulation of abnormal AAT should be assessed in Pi∗Z carriers with ACLD.

## Graphical abstract

**Figure:**
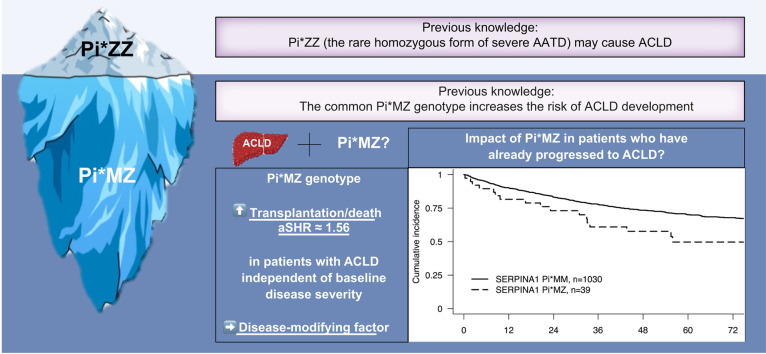

## Introduction

Alpha-1 antitrypsin deficiency (AATD) is a genetic cause of chronic liver disease.^1^ Pathophysiologically, accumulation of misfolded polymerized alpha-1 antitrypsin protein within the endoplasmic reticulum (ER) of hepatocytes is considered the main mechanism (*i.e*., toxic gain-of-function/proteotoxicity) promoting liver disease in these patients.[2], [3], [4] In contrast, the deficiency of circulating alpha-1 antitrypsin (*i.e*., loss-of-function) is the central mechanism underlying emphysema formation, which is the other major manifestation of AATD. While Pi∗M constitutes the wild-type allele, common deficiency alleles are Pi∗Z and Pi∗S, the latter leading to a less severe form of AATD. Early autopsy studies suggested that about one-third of patients homozygous for the Pi∗Z allele (serpin family A member 1 [*SERPINA1*] rs28929474[T]) develop cirrhosis.^2^ Recent data derived from the UK biobank^5^ and a European multicentre study^6^ indicated that liver stiffness and the probability of cirrhosis increase with the number of Pi∗Z alleles, with the highest values in patients with Pi∗ZZ followed by Pi∗SZ, and Pi∗MZ. Pi∗ZZ AATD may lead to advanced chronic liver disease (ACLD) even in the absence of additional etiological factors or cofactors, while additional forms of hepatic injury are usually required for carriers of Pi∗SZ and Pi∗MZ to progress to ACLD. Of note, a considerably higher AATD-related liver disease burden was observed in the presence of metabolic (co)factors.^7^ Individuals harbouring the Pi∗ZZ genotype have a 20-fold higher risk of liver fibrosis/cirrhosis compared to non-carriers.^8^ Analyses of the UK biobank suggested an odds ratio higher than 40 for the development of liver tumours, however, further studies are needed to clarify the role Pi∗ZZ in liver tumour development.^8^ Harbouring the Pi∗SZ variant was associated with a 3-fold and 7-fold increase in the risk of liver fibrosis/cirrhosis and liver cancer, respectively.^8^ In Pi∗MZ individuals an odds ratio of 1.7 for liver fibrosis/liver-related mortality was reported, whereas no significant risks of liver cancers could be demonstrated.[8], [9], [10]

However, despite the overrepresentation of Pi∗Z allele carriers among patients with ACLD, in particular those with higher disease severity,^11^ data on the impact of AATD-associated mutations on the clinical course of patients who have already progressed to ACLD are scarce. In a study by Schaefer *et al.*,^11^ in individuals with cirrhosis, including 488 individuals with Pi∗MM and 52 with Pi∗MZ, the Pi∗MZ genotype was associated with more advanced hepatic dysfunction and decompensation. In the subgroup of patients with hypotransferrinaemia or increased transferrin saturation, the Pi∗MZ genotype was accompanied by an increased risk of liver transplantation/death during follow-up, however, there was no statistically significant association in the overall study population. Finally, Pi∗MS showed no association with liver disease severity.^11^

The aim of our study was to evaluate the impact of the *SERPINA1* Pi∗Z allele on liver-related death or the requirement for liver transplantation, while adjusting for the severity of liver disease at baseline in a large, thoroughly characterized cohort of patients with ACLD who underwent hepatic venous pressure gradient (HVPG) measurement. Moreover, we aimed to investigate the implications of *SERPINA1* Pi∗S allele on the course of liver disease.

## Patients and methods

### Study design and patients

We performed a retrospective, single-centre cohort study in patients with ACLD who underwent HVPG measurement at the Vienna Hepatic Hemodynamic Lab. Inclusion criteria were (i) liver stiffness measurement ≥10 kPa and/or HVPG ≥6 mmHg, (ii) valid HVPG measurement, and (iii) availability of information on *SERPINA1* genotype. Furthermore, patients were excluded if any of the following criteria were present: Patients with a history of orthotopic liver transplantation, any active extrahepatic malignancy, patients with non-parenchymal liver diseases, or missing information on important laboratory parameters and/or clinical follow-up. Patients were included between Q2/04 and Q4/20 and recruitment and follow-up over the study period was depicted in Fig. S1. Study-relevant clinical and laboratory information was collected from patients’ medical records.

### HVPG measurement

Under local anaesthesia and ultrasound guidance, a catheter introducer sheath was inserted into the right internal jugular vein. Subsequently, a hepatic vein was cannulated and the free and wedged hepatic venous pressures were obtained at least as triplicate measurements^12^ by a balloon catheter.^13^

### Genotyping

After blood collection of EDTA blood (4 ml), the samples were frozen to -20°C. Prior to DNA isolation, which took place within days to weeks after blood collection, blood was thawed to room temperature. Thereafter, genomic DNA was isolated from 200 μl of blood using the QiAmp Blood Mini Kit (QIAGEN N.V., Hilden, Germany). The optical density of DNA was then measured (DNA content and purity) using a NanoDrops 1000 Spectrophotometer from Thermo Fisher (Thermo Fisher Scientific Inc., Waltham, MC, US) and 1 μl (equivalent to approximately 30 nanograms) of this DNA was analysed by real-time PCR (7500 Fast Real-Time PCR System, TaqMan SNP Genotyping Assay [Applied Biosystems, Foster City, CA, US]).

### Reporting of ethnicity

Ethnicity was determined based on geographic origin and data is presented in accordance with recently published guidelines on reporting ethnicity in research articles.^14^

### Statistical analysis

All statistical analyses were performed using IBM SPSS Statistics 27 (IBM, New York, NY, USA), R 4.1.2 (R Core Team, R Foundation for Statistical Computing, Vienna, Austria), or GraphPad Prism 8 (GraphPad Software, CA, USA). Categorical variables were reported as absolute (n) and relative frequencies (%), whereas continuous variables as mean ± SD or median (IQR), as appropriate. Student’s *t* test was used for group comparisons of normally distributed variables and Mann-Whitney *U* test for non-normally distributed variables. Group comparisons of categorical variables were performed using either Chi-squared or Fisher’s exact test, as appropriate.

Follow-up time was calculated as the time from HVPG measurement to the date of liver transplantation, death, or last follow-up at one of the hospitals of the Vienna hospital association by the reverse Kaplan-Meier method.

The impact of *SERPINA1* single nucleotide variants on liver-related death or the requirement for liver transplantation was assessed using competing risk analysis considering non-liver-related death, or date of removal/suppression of the primary etiological factor (initiation of antiviral therapy/reported alcohol abstinence), as defined by Baveno VII,^15^ as competing risks. Therefore, Fine and Gray competing risks regression models (cmprsk: subdistribution analysis of competing risks, https://CRAN.R-project.org/package=cmprsk)^16^ were calculated. Baseline characteristics that were significantly different between patients with or without the respective *SERPINA1* single nucleotide variants or which we considered of particular importance for the endpoint of interest (*i.e*., age, indicators of hepatic dysfunction, and HVPG; patatin-like phospholipase domain containing 3 *[PNPLA3]* GG genotype in an additional analysis) were included into the multivariable competing risk model as covariables. The Child-Turcotte-Pugh (CTP) and United Network for Organ Sharing (UNOS) model for end-stage liver disease (MELD) (2016) scores have significant overlap in terms of included variables. Therefore, we generated separate models with either CTP or UNOS MELD (2016) scores. To confirm our findings without the consideration of competing risks, we have additionally calculated Cox regression models and included them into the supplementary information.

The level of significance was set at a 2-sided *p* value <0.05.

### Ethics

The study has been conducted in accordance with the principles of the Declaration of Helsinki and its amendments and has been approved by the local ethics committee, which waived the requirement for written informed consent. However, all patients signed a written informed consent for genetic testing.

## Results

### Study population

**Fig. 1:**
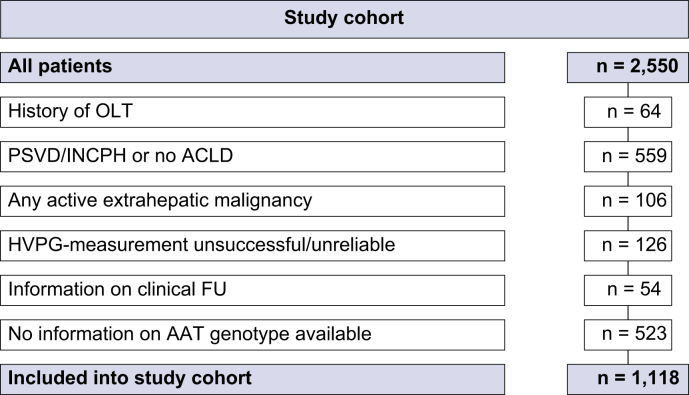
Study flowchart. AAT, alpha-1 antitrypsin; ACLD, advanced chronic liver disease; FU, follow-up; HVPG, hepatic venous pressure gradient; INCPH, idiopathic non-cirrhotic portal hypertension; PSVD, porto-sinusoidal vascular disease.

Overall, 2,550 patients underwent HVPG measurement within the study period (Fig. 1). After applying inclusion and exclusion criteria, 1,118 patients were finally included in our study. Mean age was 55±12 years and most patients were male (n = 776, 69%; Table 1). Viral hepatitis was the leading etiology of liver disease (n = 495, 44%), followed by alcohol-related liver disease (n = 351, 31%), non-alcoholic fatty liver disease (NAFLD; n = 121, 11%), and other liver disease etiologies (n = 151, 14%). Regarding portal hypertension severity, mean HVPG was 15±7 mmHg and 62% of patients (n = 577) had varices, of whom 135 (12%) had a history of variceal bleeding. Mean UNOS MELD (2016) was 12±5 points and mean CTP score was 7±2 points. Almost half of patients had already experienced decompensation at study inclusion (n = 509, 46%), and 13% of patients (n = 149) had been diagnosed with HCC.

**Table 1:** **Comparison of patient characteristics according to the *SERPINA1* rs28929474 genotype/Pi∗Z allele**.

Patient characteristics	*SERPINA1* rs28929474			
	All patients n = 1,118	Pi∗Z non-carriers^1^ G;G n = 1,076 (96%)	Pi∗Z carriers^2^ A;G n = 40 (4%); A;A n = 2 (0.2%)	*p* value
Age, years, mean ± SD	55.0 ± 12.0	54.9 ± 12.0	57.8 ± 11.9	0.124
Sex, n (%)				
Male	776 (69%)	754 (69%)	31 (74%)	0.611
Female	342 (31%)	331 (31%)	11 (26%)	
Etiology, n (%)				
ALD	351 (31%)	338 (31%)	13 (31%)	**0.012**
NAFLD	121 (11%)	111 (10%)	10 (24%)	
Viral	495 (44%)	484 (45%)	11 (26%)	
Other	151 (14%)	143 (13%)	8 (19%)	
HVPG, mmHg, mean ± SD	15 ± 7	15 ± 7	19 ± 6	**<0.001**
UNOS MELD (2016) score, point, mean ± SD	12 ± 5	12 ± 5	13 ± 5	0.161
CTP score, mean ± SD	6.5 ± 1.9	6.5 ± 1.9	7.1 ± 1.9	**0.050**
A, n (%)	694 (62%)	674 (63%)	20 (48%)	0.143
B, n (%)	324 (29%)	307 (29%)	17 (41%)	
C, n (%)	100 (9%)	95 (9%)	5 (12%)	
Varices, n (%)	577 (62%)	548 (62%)	29 (76%)	0.194
History of variceal bleeding, n (%)	135 (12%)	126 (12%)	9 (21%)	0.058
Decompensated, n (%)	509 (46%)	483 (45%)	26 (62%)	**0.030**
HCC, n (%)	149 (13%)	146 (14%)	3 (7%)	0.229
Sodium, mmol x L^−1^, mean ± SD	138.0 ± 3.5	138.0 ± 3.5	137.6 ± 3.4	0.450
Creatinine, mg x dl^−1^, median (IQR)	0.8 (0.7–0.9)	0.8 (0.7–0.9)	0.7 (0.7–0.8)	0.128
Bilirubin, mg x dl^−1^, median (IQR)	1.1 (0.7–1.8)	1.0 (0.7–1.8)	1.3 (1.0–2.1)	**0.010**
Albumin, g x L^−1^, mean ± SD	36.4 ± 5.8	36.5 ± 5.8	34.5 ± 5.7	**0.032**
CRP, mg x L^−1^, median (IQR)	0.3 (0.1–0.7)	0.3 (0.1–0.7)	0.3 (0.1–1.0)	0.280
INR, mean ± SD	1.3 ± 0.3	1.3 ± 0.3	1.4 ± 0.3	0.075
AST, U x L^−1^, median (IQR)	52 (35–79)	52 (35–80)	55 (38–72)	0.411
ALT, U x L^−1^, median (IQR)	38 (24–68)	38 (24–69)	36 (27–51)	0.526
GGT, U x L^−1^, median (IQR)	105 (57–185)	104 (57–185)	140 (55–190)	0.502

Values in bold designate *p* values <0.05. ALD, alcohol-related liver disease; ALT, alanine aminotransferase; AST, aspartate aminotransferase; CRP, C-reactive protein; CTP, Child-Turcotte-Pugh; GGT, gamma-glutamyl transferase; HCC, hepatocellular carcinoma; HVPG, hepatic venous pressure gradient; INR, international normalized ratio; NAFLD, non-alcoholic fatty liver disease; UNOS MELD (2016), United Network for Organ Sharing model for end-stage liver disease (2016) score.

1 Pi∗MM n = 1,030, Pi∗MS n = 45, and Pi∗SS n = 1.

2 Pi∗MZ n = 39, Pi∗ZZ n = 2, and Pi∗SZ n = 1.

Overall, 1,030 patients (92%) harboured the wild-type (Pi∗MM), whereas 40 patients had one (Pi∗MZ n = 39; 4%; Pi∗SZ n = 1; 0.1%) and two patients had two Pi∗Z alleles (Pi∗ZZ; 0.2%). Moreover, 46 patients (4%) had one (Pi∗MS, one patient Pi∗SZ) and one patient two Pi∗S alleles (Pi∗SS; 0.1%). Due to the low number of homozygous Pi∗Z/Pi∗S allele carriers, we decided to group patients with one and two Pi∗Z/Pi∗S alleles for statistical analyses (n = 42, 4%; n = 47, 4%). Most included patients were of European descent (n = 1,029, 92%), followed by patients from Arabic (n = 69, 6%), Asian (n = 16, 1.4%) and African descent (n = 4, 0.4%). Of note, all Pi∗Z and Pi∗S carriers were of European descent.

### Patient characteristics according to *SERPINA1* rs28929474 genotype/the Pi∗Z allele

At baseline, Pi∗Z carriers were slightly older (57.8 ± 11.9 *vs.* 54.9 ± 12.0 years; *p =* 0.124) and more commonly had NAFLD than patients without Pi∗Z (24% *vs.* 10%; *p =* 0.012), while viral hepatitis was comparatively uncommon (26% *vs.* 45%; *p =* 0.012; Table 1). HVPG was significantly higher in Pi∗Z carriers (19 ± 6 *vs.* 15 ± 7 mmHg; *p <*0.001), with an increased (though not statistically significant) proportion of Pi∗Z carriers having a history of variceal bleeding (21% *vs.* 12%; *p =* 0.058). Moreover, a higher number of patients had already experienced decompensation (62% *vs.* 45%; *p =* 0.030) and CTP score tended to be higher in Pi∗Z carriers (7.1 ± 1.9 *vs.* 6.5 ± 1.9 points; *p =* 0.050).

### Impact of *SERPINA1* rs28929474 genotype/the Pi∗Z allele on risk of liver transplantation/liver-related death

Median follow-up time was 64.6 (95% CI 59.4-70.9) months. Overall, 319 patients (28.5%) achieved etiological cure and 100 patients (8.9%) underwent liver transplantation. During the study period, 377 patients (33.7%) died and 297 of these deaths were considered liver related.

**Fig. 2:**
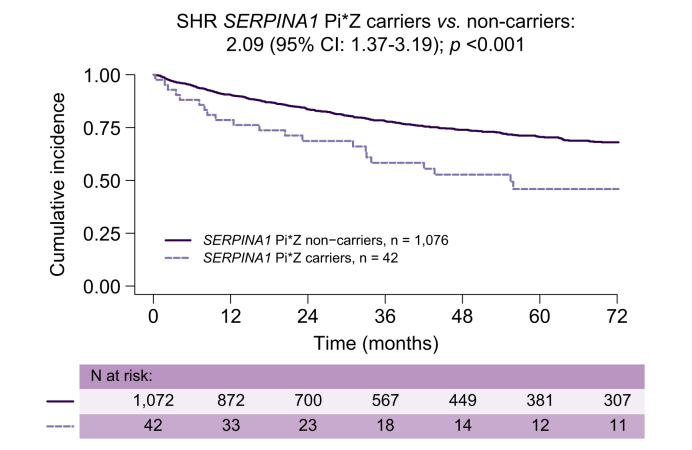
Cumulative incidences of liver transplantation/liver-related death in *SERPINA1* Pi∗Z carriers *vs.* non-carriers with etiological cure and non-liver-related death as competing risks. SHR, subdistribution hazard ratio.

Fig. 2 shows the cumulative incidences of liver transplantation/liver-related death stratified according to the presence of the Pi∗Z allele. In competing risk regression analysis, harbouring the Pi∗Z allele was significantly associated with liver-related death or the requirement for liver transplantation in univariable analysis (subdistribution hazard ratio [SHR] 2.09; 95% CI 1.37-3.19; *p <*0.001). Importantly, this result was confirmed (model 1: adjusted SHR [aSHR] 1.75; 95% CI 1.15-2.65; *p =* 0.009) after adjustment for age, HVPG, and CTP stage, as well as for age, HVPG, UNOS MELD (2016), and decompensation status (model 2: aSHR 1.80; 95% CI 1.18-2.74; *p =* 0.007; Table 2).

**Table 2:** **Multivariable competing risk regression analysis of risk of liver transplantation/liver-related death, with etiological cure and non-liver-related death as competing risks**.

Patient characteristics	Model 1		Model 2	
	aSHR (95%CI)	*p* value	aSHR (95%CI)	*p* value
Age, year	1.03 (1.02–1.04)	<0.001	1.03 (1.02–1.04)	<0.001
HVPG, mmHg	1.03 (1.01–1.04)	0.004	1.03 (1.01–1.05)	0.003
CTP stage A	1	—	—	—
CTP stage B	2.00 (1.52–2.62)	<0.001	—	—
CTP stage C	4.24 (2.91–6.17)	<0.001	—	—
UNOS MELD (2016) score, point	—	—	1.09 (1.06–1.12)	<0.001
dACLD	—	—	1.10 (0.84–1.43)	0.500
*SERPINA1* Z allele	1.75 (1.15–2.65)	0.009	1.80 (1.18–2.74)	0.007

aSHR, adjusted subdistribution hazard ratio; CTP, Child-Turcotte-Pugh score; dACLD, decompensated advanced chronic liver disease; HVPG, hepatic venous pressure gradient; UNOS MELD (2016), United Network for Organ Sharing model for end-stage liver disease (2016) score.

When only considering patients with the Pi∗MM (n = 1,030) and the Pi∗MZ (n = 39; *i.e*., the common heterozygous form of AAT-related liver disease) genotypes, the Pi∗MZ genotype was associated with liver-related death or the requirement for liver transplantation in univariable analysis (SHR 1.88; 95% CI 1.20-2.95; *p =* 0.006; Fig. S2). Importantly, this effect was maintained in multivariable analyses (model 1: aSHR 1.56; 95% CI 1.01-2.41; *p =* 0.047; model 2: aSHR 1.61; 95% CI 1.03-2.51; *p =* 0.036; Table S1). Results were confirmed in Cox regression analyses as depicted in Tables S2 and S3.

### Impact of *SERPINA1* rs28929474 genotype/the Pi∗Z allele on risk of liver transplantation/liver-related death in different etiologies of liver disease

Detailed information is provided in the Fig. S3.

### Impact of the Pi∗Z allele when accounting for *PNPLA3* GG genotype

The distribution of *PNPLA3* genotypes (CC: n = 489 [44%], GC: n = 464 [41%], GG: n = 165 [15%]) was similar compared to previously published data from a partly overlapping cohort.^17^ Harbouring the *PNPLA3* GG genotype was associated with an increased risk of liver transplantation/liver-related death (HR 1.44; 95% CI 1.11-1.88; *p =* 0.007) in univariable analysis**.** Interestingly, the impact of the Pi∗Z allele was independent of the *PNPLA3* GG genotype (Table S4).

### Impact of *SERPINA1* rs17580 genotype/the Pi∗S allele on risk of liver transplantation/liver-related death

Next, we evaluated the impact of the Pi∗S allele on the clinical course of patients who had already progressed to ACLD. When comparing patients with Pi∗S allele(s) to non-carriers, no significant differences were observed at study inclusion (Table S5).

Fig. S4 depicts the cumulative incidence plot of liver transplantation/liver-related death in patients with *vs.* without (the) Pi∗S allele(s). There was no difference in the risk of liver transplantation/liver-related death between Pi∗S carriers (SHR 1.13; 95% CI 0.69-1.86; *p =* 0.630) *vs.* non-carriers. This finding was confirmed when adjusting for covariables, as demonstrated in Table S6.

## Discussion

Liver transplantation is currently the only established treatment option for severe AATD-related liver disease.^18^ Our study provides evidence of the significance of the Pi∗Z allele and associated deficiency genotypes Pi∗MZ (and Pi∗SZ and Pi∗ZZ) on disease progression beyond the development of ACLD, suggesting that pharmacological interventions targeting the toxic gain-of-function by decreasing the production or increasing the degradation of Z protein have the potential to ameliorate liver disease progression in patients with ACLD.

In an unselected cohort of patients undergoing HVPG measurement, we demonstrated that Pi∗Z carriage was not very common in patients with ACLD (4%). This is less frequent than in comparable studies (Schaefer *et al.* [9.6%],^11^ Strnad *et al.* [13.8%]^19^), which may be explained by the high proportion of patients with viral etiology in our study. In these patients, Pi∗Z carriage seemed to be less detrimental, as indicated by a numerically lower SHR for liver transplantation/liver-related death, compared to other etiologies. Besides having more severe portal hypertension compared to non-carriers, Pi∗Z carriers also had more advanced hepatic dysfunction at the time of evaluation. Contrarily, the Pi∗S allele showed no significant differences among included patients, indicating that – in the absence of the Z allele – it is unrelated to liver disease and its progression in patients with ACLD. This is in line with a population-based study investigating its potential impact on the progression to cirrhosis.^8^ Considering its association with liver transplantation/liver-related death, carriers of the Pi∗Z allele were at increased risk, even after multivariable adjustment. This is in accordance with data from the UK Biobank^8^ and a Finnish population-based study,^9^ indicating an increased risk of liver-related mortality. Of note, this is the first study adjusting for portal hypertension severity (*i.e.*, HVPG) at baseline and thereby accounting for an additional important prognostic indicator. Since the mean UNOS MELD (2016) of our study population was low (*i.e.*, 12 points), and thus, most patients had no indication for liver transplantation at baseline, we included the requirement for liver transplantation (together with death) in a composite endpoint, rather than analysing liver transplantation as a competing event. Regarding the impact of Pi∗Z in different etiologies of liver disease, the question arises whether heterozygosity for the Pi∗Z allele is considered solely as a disease modifier or whether AATD may even be seen as the primary etiological factor in some of these patients. Interestingly, patients with viral hepatitis (*i.e*., those with the most evident primary etiological factor) were underrepresented among Pi∗Z carriers, while NAFLD was overrepresented. This is in line with previous studies reporting a more consistent association between Pi∗MZ genotype and ACLD in patients with fatty liver disease, as compared to other common etiologies.^5^ One explanation may be that NAFLD – which was not always biopsy-proven – may have been misdiagnosed. However, the newly proposed MAFLD term also accounts for the concept that genetic predisposition *vs.* environmental factors *vs.* metabolic syndrome vary on a case-by-case basis. However, the overrepresentation of NAFLD etiology among patients harbouring the Pi∗Z allele may also be explained by the interaction between the metabolic syndrome and the Pi∗Z allele, as individuals with Pi∗ZZ and metabolic syndrome showed more accumulated abnormal AAT in hepatocytes,^20^ indicating that the presence of metabolic syndrome – which overlaps with NAFLD – may amplify the detrimental impact of the Pi∗Z variant. However, in univariable analyses, we observed numerically increased risks of liver transplantation/death within all included etiologies. Even so, sample size/statistical power was limited when sub-stratifying patients according to disease etiology and did not allow for adjustment for baseline disease severity. Accordingly, no firm conclusions can be drawn from this etiology-specific data. Moreover, due to the role of the Pi∗Z allele as a disease-modifying factor/a risk factor for disease progression, the question arises whether the removal/suppression of the primary etiological factor (*i.e*., viral eradication/suppression in HCV/HBV or alcohol abstinence) is similarly beneficial in these patients, or whether the genetic background hinders disease regression/promotes progressive disease. Although a recent study found no impact of other genetic variants in this context, the specific role of the Pi∗Z allele may be addressed by future studies.

Recently, the Pi∗MZ genotype has been associated with hepatic decompensation, the requirement for liver transplantation and liver-related death in a cohort of patients with compensated ACLD^21^; NAFLD was the main etiology (45%) and the prevalence of obesity (median BMI >30 kg x m^-2^) and metabolic comorbidities (*e.g*., diabetes prevalence ∼50%) was high. In this cohort, n = 49/574 (9%) were Pi∗Z allele carriers, compared to 3.8% in our study. This may be explained by the high prevalence of NAFLD in the US cohort, as patients with NAFLD included in our study also more commonly harboured the Pi∗Z allele. Importantly, the metabolic syndrome (MetS) has been shown to fuel AATD-related liver disease, as both periodic-acid-Schiff (PAS)-positive diastase-resistant globules in hepatocytes and liver fibrosis were more common in MetS. Accordingly, it cannot be assumed that Pi∗Z allele carriage has the same impact on liver phenotype and the development of liver-related clinical events in patients with and without MetS/NAFLD. Furthermore, the analyses were not adjusted for portal hypertension severity (*i.e.*, HVPG), which differed across genotypes in our study and drives the development of first hepatic decompensation.^22^ Therefore, our study provides important data that confirms the significance of heterozygosity of this genetic variant on disease progression in a European cohort. The same considerations apply when comparing our work to the Schaefer *et al.*^11^ study, which did not account for portal hypertension at the time of study inclusion and only showed a trend towards an increase in liver-related death or requirement for liver transplantation in patients harbouring the Pi∗MZ genotype.

Of note, the observed aSHR for Pi∗MZ *vs.* Pi∗MM (≈1.56) was similar to the adjusted hazard ratio (1.42) for *PNPLA3* rs738409 GG (*i.e*., homozygosity for the minor/risk G allele) in a previous smaller study^17^ performed in a partly overlapping patient cohort. Although the prevalence – and thus, effect on a population level – of the *PNPLA3* GG genotype was higher (15%) compared to Pi∗MZ (4%), it is important to note that the consequence of the Pi∗Z allele, *i.e.*, the hepatic Pi∗Z accumulation, is druggable.^5^^,^^23^ This is an important difference to *PNPLA3* – which is not druggable – as our observation regarding the disease-modifying effect of Pi∗Z allele may even have therapeutic implications in the future. Notably, the association between the Pi∗Z allele and the outcome of interest was confirmed in an analysis accounting for *PNPLA3* GG genotype.

The anti-protease AAT, mainly expressed in hepatocytes and secreted into the bloodstream, protects the lungs from proteolytic degradation by neutrophil elastase.^3^ In AATD-related liver disease, AAT is misfolded and intracellular polymerized, resulting in enhanced protein degradation and/or aggregation in the ER of hepatocytes, generating proteotoxic stress and hepatocellular injury. The resulting ER stress and/or environmental triggers stimulate AAT production, thereby causing a vicious cycle.^20^ Therapeutic approaches for AATD-related liver disease include RNA interference to decrease AAT production and secretion, and autophagy enhancers to reduce protein accumulation. Finally, polymerization inhibitors may facilitate both secretion and degradation.^5^ Recently, the small-interfering RNA fazirsiran (previously known as ARO-AAT) showed encouraging results. In the ARO-AAT2002 open-label trial (NCT03946449), fazirsiran was administered to patients with Pi∗ZZ and patients were biopsied after 24 or 48 weeks. The small-interfering RNA treatment resulted in marked reductions in hepatic Pi∗Z levels. Furthermore, liver function parameters (*i.e.*, alanine aminotransferase and gamma-glutamyltransferase) decreased after ARO-AAT application. Interestingly, regression of fibrosis was observed in 6 out of the 9 included participants, including two individuals with cirrhosis at study inclusion.^23^ The publication of phase II data on fazirsiran for AATD-related liver disease in the *New Engl J Med*^24^ denotes a therapeutic breakthrough, which will likely promote interest/progress in this field.^24^ While fazirsiran does not seem to be a viable option for Pi∗MZ patients due to the risk of inducing lung disease, there are several other approaches (recently reviewed in^5^) that may be used in the comparatively common Pi∗MZ genotype. Besides fazirsiran^24^ and comparable investigational compounds (NCT04174118) – or in the future possibly even Pi∗Z-specific RNA inhibition – and autophagy-inducing drugs (*e.g*., carbamazepine is currently being evaluated in a clinical trial [NCT01379469] and nor-ursodeoxycholic acid which has shown promise in preclinical studies^25^), there are also ongoing clinical trials on folding correctors.^5^ Accordingly, the development pipeline of treatments for AATD-related liver disease is expanding and includes agents that may be suitable for being evaluated in the context of Pi∗MZ-associated ACLD. However, with a prevalence of ≈1:2,000, Pi∗ZZ carriers are only the tip of the AATD iceberg, which primarily comprises Pi∗MZ carriers (prevalence: ≈1:30).^5^ The main finding of our study, *i.e*., the detrimental impact of Pi∗MZ on the course of ACLD, suggests that treatments targeting the Z protein, such as fazirsiran may be evaluated as disease-modifying therapies in patients with ACLD, *i.e.*, those with the most urgent need for effective therapies. Of note, our study is the first to indicate significantly increased rates of liver transplantation/liver-related death when comparing patients with *vs.* without the Pi∗Z allele and established ACLD. Until specific treatments become available, carriage of Pi∗Z should be considered as a genetic risk factor for disease progression beyond the development of ACLD.

The main limitation of our study is its retrospective design. However, patients were thoroughly characterized at the time of HVPG measurement. The assessment of the portal hypertension severity (*i.e*., HVPG measurement) is an important strength of our study, compared to previous longitudinal studies^9^^,^^11^ which did not account for portal hypertension severity at baseline. Since patients were included during HVPG measurement, we cannot rule-out selection bias. However, a high number of HVPG measurements are performed at our institution for risk stratification and treatment monitoring purposes.^12^ Other studies investigating the impact of Pi∗Z allele on liver diseases were restricted to specific liver disease etiologies or genotypes, which was not the case for our study, as we aimed to maximize sample size. In this context, we have merged all carriers of the Pi∗Z allele (of whom n = 39/42 were Pi∗MZ) for the main analysis, however, the impact of the Pi∗MZ (n = 39; as compared to the Pi∗MM, n = 1,030) genotype was confirmed in a subgroup analysis. Of note, merging all carriers of the Pi∗Z allele (*i.e.*, not considering the Pi∗S allele) in the main analysis allows for direct comparisons with other studies investigating risk/protective alleles (*i.e.*, *PNPLA3*^17^ and *HSD17B13*^26^), while this would have not been possible when analysing only haplotypes (*i.e*., the Pi∗Z and Pi∗S allele). Of note, the findings of our study may only apply to individuals of European descent, as only a small number of those of non-European descent were included. Although we cannot formally rule-out overlap between the patient populations of Schaefer *et al.*’s study^11^ and the present study, this seems extremely unlikely, given the high geographical distance (liver transplant centres for the East/West of Austria) between the recruiting centres. Finally, our study did not include a validation cohort, as we are not aware of another adequately sized cohort linking information on HVPG and genetic data.

In conclusion, we demonstrate the profound detrimental impact of the Pi∗Z allele on the outcome of ACLD. Genotyping for the Pi∗Z allele identifies patients at increased risk, thereby improving prognostication. Finally, the role of medical therapies targeting the accumulation of abnormal AAT should be evaluated as disease-modifying treatments in Pi∗Z allele carriers with ACLD.

## Financial support

No specific financial support was received for this study.

## Authors’ contributions

Concept of the study (L.B., B.Sc., and M.M.), data collection (L.B., B.Sc., M.U., P.W., R.P., B.Si., L.H., and M.J.), statistical analysis (L.B., B.Sc., and M.M.), drafting of the manuscript (L.B., B.Sc., and M.M.), and revision for important intellectual content and approval of the final manuscript (all authors).

## Data availability statement

The data that support the findings of this study are available from the corresponding author upon reasonable request.

## Conflicts of interest

The authors have nothing to disclose regarding the work under consideration for publication. Conflicts of interests outside the submitted work: L.B., M.U., P.W., R.P., L.H., M.J., G.S., C.W., P.F., and A.F.S. have nothing to disclose. B.Sc. Received travel support from AbbVie, Ipsen, and Gilead. B.Si. Received travel support from AbbVie and Gilead. D.B. received speaker fees from AbbVie and Siemens, as well as grant support form Gilead and Siemens, as well as travel support from AbbVie and Gilead. M.P. served as a speaker and/or consultant and/or advisory board member for Bayer, Bristol-Myers Squibb, Eisai, Ipsen, Lilly, MSD, and Roche, and received travel support from Bayer and Bristol-Myers Squibb. P.F. served as a speaker and/or consultant and/or advisory board member for AbbVie, Gilead, MYR Pharmaceuticals, and Vivaraxx and received grants/research support from Gilead. M.T. served as a speaker and/or consultant and/or advisory board member for Albireo, BiomX, Falk, Boehringer Ingelheim, Bristol-Myers Squibb, Falk, Genfit, Gilead, Intercept, Janssen, MSD, Novartis, Phenex, Pliant, Regulus, and Shire, and received travel support from AbbVie, Falk, Gilead, and Intercept, as well as grants/research support from Albireo, Alnylam, Cymabay, Falk, Gilead, Intercept, MSD, Takeda, and UltraGenyx. He is also co-inventor of patents on the medical use of 24-norursodeoxycholic acid. T.R. served as a speaker and/or consultant and/or advisory board member for AbbVie, Bayer, Boehringer Ingelheim, Gilead, Intercept, MSD, Siemens, and W. L. Gore & Associates and received grants/research support from AbbVie, Boehringer Ingelheim, Gilead, Intercept, MSD, Myr Pharmaceuticals, Pliant, Philips, Siemens, and W. L. Gore & Associates as well as travel support from AbbVie, Boehringer Ingelheim, Gilead and Roche. M.M. served as a speaker and/or consultant and/or advisory board member for AbbVie, Gilead, and W. L. Gore & Associates and received travel support from AbbVie and Gilead.

Please refer to the accompanying ICMJE disclosure forms for further details.
