# Supplementary material for: Alpha-1 antitrypsin Pi∗Z allele is an independent risk factor for liver transplantation and death in patients with advanced chronic liver disease
Source: JHEP Rep. 2022 Aug 20;4(11):100562. doi: 10.1016/j.jhepr.2022.100562 (PMC9513767; doi:10.1016/j.jhepr.2022.100562)
Supplement: Multimedia component 1 [file mmc1.pdf]

Alpha-1 antitrypsin Pi*Z allele is an independent risk factor for liver transplantation and death in patients with advanced chronic liver disease

Lorenz Balcar, Bernhard Scheiner, Markus Urheu, Patrick Weinberger, Rafael Paternostro, Benedikt Simbrunner, Lukas Hartl, Mathias Jachs, David Bauer, Georg Semmler, Claudia Willheim, Matthias Pinter, Peter Ferenci, Michael Trauner, Thomas Reiberger, Albert Friedrich Stättermayer, Mattias Mandorfer

Table of contents

Supplementary results 2
Fig. S1 3
Fig. S2 4
Fig. S3 5
Fig. S4 6
Table S1 7
Table S2 9
Table S3 10
Table S4 10
Table S5 12
Table S6 14

Supplementary results

Impact of *SERPINA1* rs28929474 genotype/the Pi*Z allele on requirement of liver transplantation/liver-related death in different etiologies of liver disease

When stratifying patients according to their etiology of liver disease, there was a trend towards a positive association with liver transplantation/liver-related death in those harbouring the Pi*Z allele among patients with fatty liver disease (n=23/472 with Pi*Z allele; SHR: 1.59 [95%CI: 0.92-2.75]; p=0.096; Fig. S2A). Among patients with viral hepatitis, Pi*Z carriers had a numerically higher subdistribution hazard as well (n=11/495 with Pi*Z allele; SHR: 1.41 [95%CI: 0.45-4.43]; p=0.560; Fig. S2B). Finally, in patients with other etiologies of parenchymal liver disease, harbouring the Pi*Z genotype (n=8) was significantly associated with the requirement of liver transplantation/liver-related death in competing risk regression analysis (SHR: 3.30 [95%CI: 1.65-6.59]; p<0.001; Fig. S2C).

Supplementary figures

Fig. S1

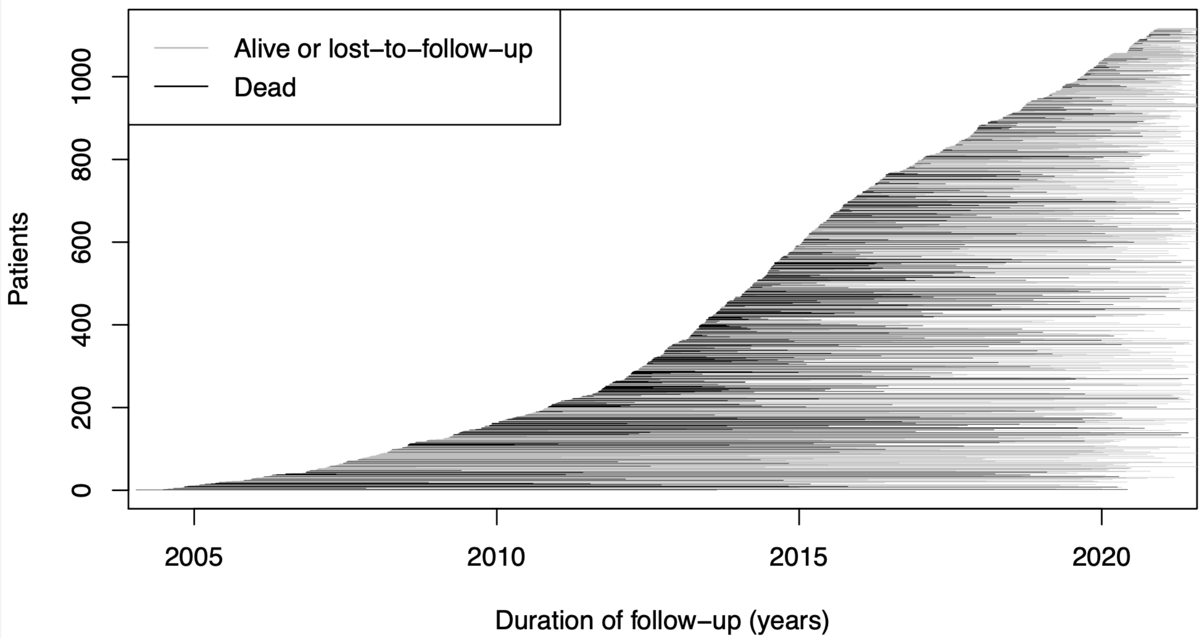

Fig. S1. Recruitment and follow-up over the study period.

Fig. S2

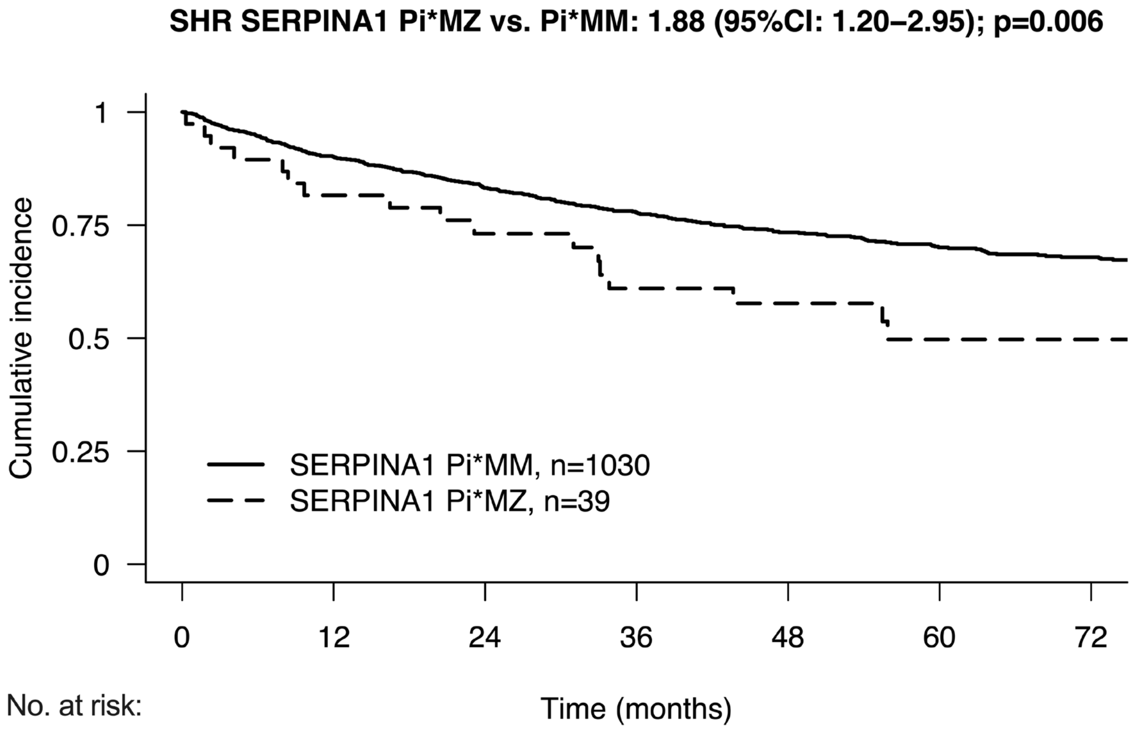

No. at risk:

——	1030	835	669	539	426	361	290
— —	39	31	22	17	13	11	10

Fig. S2. Cumulative incidences of requirement for liver transplantation/liver-related death in Serpin Family A Member 1 (*SERPINA1*) Pi*MZ vs. Pi*MM carriers with etiological cure and non-liver-related death as competing risks.

Abbreviations: SHR – subdistribution hazard ratio

Fig. S3

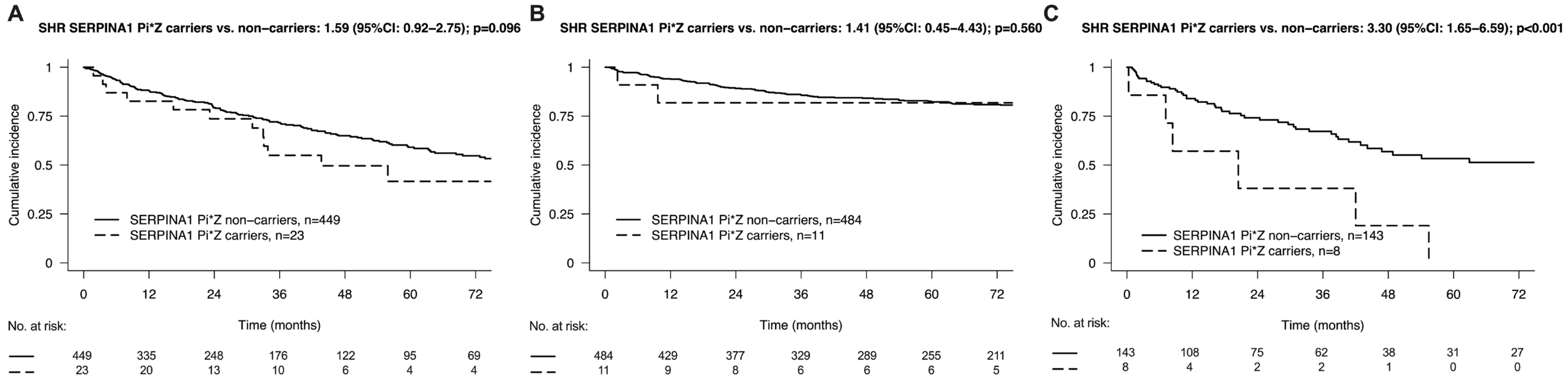

Fig. S3. Cumulative incidences of liver transplantation/liver-related death stratified in Serpin Family A Member 1 (*SERPINA1*) Pi*Z carriers vs. non-carriers and with etiological cure and non-liver-related death as competing risks, in NAFLD/ALD (**A**), viral hepatitis (**B**), and other etiologies of liver disease (**C**).

Abbreviations: SHR – subdistribution hazard ratio.

Fig. S4

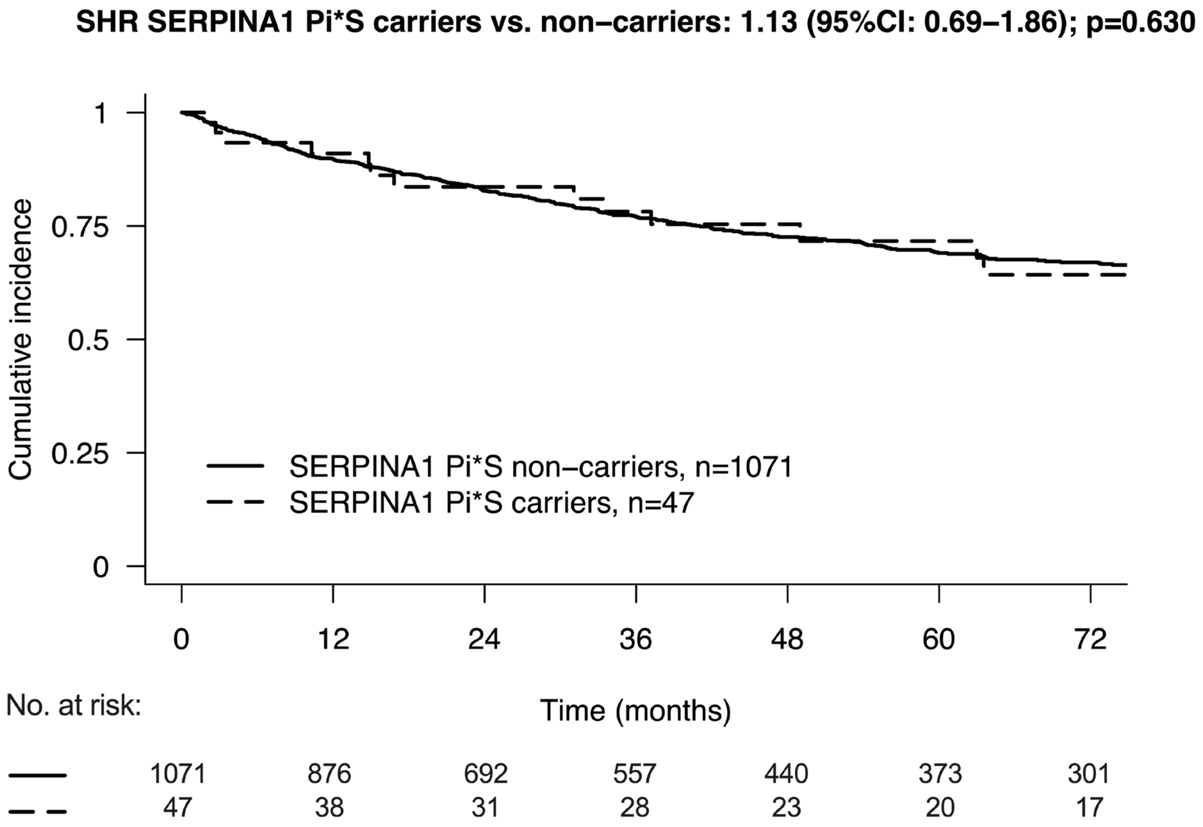

Fig. S4. Cumulative incidences of requirement for liver transplantation/liver-related death in Serpin Family A Member 1 (*SERPINA1*) Pi*S carriers vs. non-carriers with etiological cure and non-liver-related death as competing risks.

Abbreviations: SHR – subdistribution hazard ratio

SUPPLEMENTARY TABLES

Table S1

Patient characteristics	**Model 1**		**Model 2**	
	**aSHR (95%CI)**	**p-value**	**aSHR (95%CI)**	**p-value**
Age, year	1.03 (1.02-1.04)	**<0.001**	1.03 (1.02-1.04)	**<0.001**
HVPG, mmHg	1.03 (1.01-1.04)	**0.003**	1.03 (1.01-1.05)	**0.003**
CTP stage A	1	-	-	-
CTP stage B	1.99 (1.51-2.62)	**<0.001**	-	-
CTP stage C	4.27 (2.90-6.29)	**<0.001**	-	-
UNOS MELD (2016) score, point	-	-	1.09 (1.06-1.12)	**<0.001**
dACLD	-	-	1.09 (0.83-1.43)	0.520
SERPINA1 Pi*MZ vs. Pi*MM	1.56 (1.01-2.41)	**0.047**	1.61 (1.03-2.51)	**0.036**

Table S1. Multivariable competing risk regression analysis for requirement of liver transplantation or liver-related death including – among other parameters – CTP score **(model 1)** or UNOS MELD (2016)-score and dACLD **(model 2)** with etiological cure and non-liver-related death as competing risks only including Pi*MM and Pi*MZ carriers.

Abbreviations: aSHR – adjusted subdistribution hazard ratio; CTP – Child-Turcotte-Pugh score; dACLD – decompensated advanced chronic liver disease; HVPG – hepatic venous pressure gradient; UNOS MELD (2016) – United Network for Organ Sharing model for end-stage liver disease (2016) score

Table S2

Patient characteristics	**Model 1**		**Model 2**	
	**aHR (95%CI)**	**p-value**	**aHR (95%CI)**	**p-value**
Age, year	1.03 (1.02-1.04)	**<0.001**	1.03 (1.02-1.04)	**<0.001**
HVPG, mmHg	1.04 (1.02-1.06)	**<0.001**	1.04 (1.02-1.06)	**<0.001**
CTP stage A	1	-	-	-
CTP stage B	1.75 (1.36-2.25)	**<0.001**	-	-
CTP stage C	3.53 (2.54-4.90)	**<0.001**	-	-
UNOS MELD (2016) score, point	-	-	1.08 (1.06-1.11)	**<0.001**
dACLD	-	-	1.11 (0.86-1.43)	0.421
SERPINA1 Z allele	1.82 (1.20-2.75)	**0.005**	1.87 (1.24-2.84)	**0.003**

Table S2. Multivariable Cox regression analysis for requirement of liver transplantation or liver-related death including – among other parameters – CTP score **(model 1)** or UNOS MELD (2016)-score and dACLD **(model 2)**.

Abbreviations: aSHR – adjusted hazard ratio; CTP – Child-Turcotte-Pugh score; dACLD – decompensated advanced chronic liver disease; HVPG – hepatic venous pressure gradient; UNOS MELD (2016) – United Network for Organ Sharing model for end-stage liver disease (2016) score

Table S3

Patient characteristics	**Model 1**		**Model 2**	
	**aHR (95%CI)**	**p-value**	**aHR (95%CI)**	**p-value**
Age, year	1.03 (1.02-1.04)	**<0.001**	1.03 (1.02-1.04)	**<0.001**
HVPG, mmHg	1.04 (1.03-1.06)	**<0.001**	1.04 (1.02-1.06)	**<0.001**
CTP stage A	1	-	-	-
CTP stage B	1.74 (1.35-2.25)	**<0.001**	-	-
CTP stage C	3.53 (2.52-4.93)	**<0.001**	-	-
UNOS MELD (2016) score, point	-	-	1.08 (1.06-1.11)	**<0.001**
dACLD	-	-	1.12 (0.86-1.44)	0.400
SERPINA1 Pi*MZ vs. Pi*MM	1.63 (1.05-2.53)	**0.031**	1.69 (1.08-2.63)	**0.021**

Table S3. Multivariable Cox regression analysis for requirement of liver transplantation or liver-related death including – among other parameters – CTP score **(model 1)** or UNOS MELD (2016)-score and dACLD **(model 2)** only including Pi*MM and Pi*MZ carriers.

Abbreviations: aSHR – adjusted hazard ratio; CTP – Child-Turcotte-Pugh score; dACLD – decompensated advanced chronic liver disease; HVPG – hepatic venous pressure gradient; UNOS MELD (2016) – United Network for Organ Sharing model for end-stage liver disease (2016) score

Table S4

Patient characteristics	**Model 1**		**Model 2**	
	**aHR (95%CI)**	**p-value**	**aHR (95%CI)**	**p-value**
Age, year	1.03 (1.02-1.04)	**<0.001**	1.03 (1.02-1.04)	**<0.001**
HVPG, mmHg	1.04 (1.02-1.06)	**<0.001**	1.04 (1.02-1.06)	**<0.001**
CTP stage A	1	-	-	-
CTP stage B	1.72 (1.34-2.21)	**<0.001**	-	-
CTP stage C	3.53 (2.54-4.90)	**<0.001**	-	-
UNOS MELD (2016) score, point	-	-	1.08 (1.06-1.11)	**<0.001**
dACLD	-	-	1.09 (0.85-1.41)	0.491
PNPLA3 GG genotype	1.23 (0.95-1.61)	0.123	1.29 (0.99-1.68)	0.061
SERPINA1 Z allele	1.80 (1.19-2.73)	**0.005**	1.87 (1.23-2.84)	**0.003**

Table S4. Multivariable Cox regression analysis for requirement of liver transplantation or liver-related death including – among other parameters – CTP score **(model 1)** or UNOS MELD (2016)-score and dACLD **(model 2)**.

Abbreviations: aSHR – adjusted subdistribution hazard ratio; CTP – Child-Turcotte-Pugh score; dACLD – decompensated advanced chronic liver disease; HVPG – hepatic venous pressure gradient; UNOS MELD (2016) – United Network for Organ Sharing model for end-stage liver disease (2016) score

Table S5

Patient characteristics	***SERPINA1* rs17580**			
	**All patients n=1118**	**Pi*S non-carriers A;A n=1071 (96%) [1]**	**Pi*S carriers A;T n=46 (4%) T;T n=1 (0.1%) [2]**	**p-value**
Age, years, mean ± SD	55.0±12.0	54.9±12.1	57.2±9.5	0.199
Sex, n (%)				
Male	776 (69%)	742 (69%)	34 (72%)	0.656
Female	342 (31%)	329 (31%)	13 (28%)	
Etiology, n (%)				
ALD	351 (31%)	339 (32%)	12 (26%)	0.609
NAFLD	121 (11%)	114 (11%)	7 (15%)	
Viral	495 (53%)	472 (44%)	23 (49%)	
Other	151 (14%)	146 (14%)	5 (11%)	
HVPG, mmHg, mean ± SD	15±7	15±7	15±7	0.814
UNOS MELD (2016) score, point, mean ± SD	12±5	12±5	12±4	0.959
CTP score, mean ± SD	6.5±1.9	6.5±1.9	6.4±2.0	0.671
A, n (%)	694 (62%)	664 (62%)	30 (64%)	0.826
B, n (%)	324 (29%)	312 (29%)	12 (26%)	
C, n (%)	100 (9%)	95 (9%)	5 (11%)	
Varices, n (%)	577 (62%)	549 (62%)	28 (74%)	0.306
History of variceal bleeding, n (%)	135 (12%)	130 (12%)	5 (11%)	0.757
Decompensated, n (%)	509 (46%)	488 (46%)	21 (45%)	0.905
HCC, n (%)	149 (13%)	141 (13%)	8 (17%)	0.446
Sodium, mmol x L^{-1}, mean ± SD	138.0±3.5	138.0±3.6	138.5±2.9	0.323
Creatinine, mg x dL^{-1}, median (IQR)	0.8 (0.7-0.9)	0.8 (0.7-0.9)	0.8 (0.7-1.0)	0.585
Bilirubin, mg x dL^{-1}, median (IQR)	1.1 (0.7-1.8)	1.1 (0.7-1.8)	1.3 (0.7-2.3)	0.632
Albumin, g x L^{-1}, mean ± SD	36.4±5.8	36.4±5.9	37.1±5.6	0.402
CRP, mg x L^{-1}, median (IQR)	0.3 (0.1-0.7)	0.3 (0.1-0.7)	0.3 (0.1-0.7)	0.726
INR, mean ± SD	1.3±0.3	1.3±0.3	1.3±0.3	0.749

AST, U x L^{-1}, median (IQR)	52 (35-79)	52 (35-80)	54 (38-73)	0.973
ALT, U x L^{-1}, median (IQR)	38 (24-68)	38 (24-68)	41 (27-63)	0.861
GGT, U x L^{-1}, median (IQR)	105 (57-185)	105 (57-187)	113 (62-179)	0.393

[1] Pi*MM n=1030, Pi*MZ n=39, and Pi*ZZ n=2

[2] Pi*MS n=45, Pi*SZ n=1, and Pi*SS n=1

Table S5. Comparison of patient characteristics according to the Serpin Family A Member 1 (*SERPINA1*) rs17580 genotype/Pi*S allele.

Abbreviations: ALD – alcoholic liver disease; ALT – alanine-aminotransferase; AST - aspartate-aminotransferase; CRP – C-reactive protein; CTP – Child-Turcotte-Pugh; GGT – gamma-glutamyl transferase; HCC – hepatocellular carcinoma; HVPG – hepatic venous pressure gradient; INR – international normalized ratio; NAFLD – non-alcoholic fatty liver disease; UNOS MELD (2016) – United Network for Organ Sharing model for end-stage liver disease (2016) score

Table S6

Patient characteristics	**Model 1**		**Model 2**	
	**aSHR (95%CI)**	**p-value**	**aSHR (95%CI)**	**p-value**
Age, year	1.03 (1.02-1.05)	**<0.001**	1.03 (1.02-1.04)	**<0.001**
HVPG, mmHg	1.03 (1.01-1.04)	**0.003**	1.03 (1.01-1.05)	**0.002**
CTP stage A	1	-	-	-
CTP stage B	2.03 (1.55-2.65)	**<0.001**	-	-
CTP stage C	4.19 (2.86-6.13)	**<0.001**	-	-
UNOS MELD (2016) score, point	-	-	1.09 (1.06-1.12)	**<0.001**
dACLD	-	-	1.10 (0.84-1.44)	0.480
SERPINA1 S-allele	0.97 (0.58-1.60)	0.890	0.98 (0.59-1.61)	0.920

Table S6. Multivariable competing risk regression analysis for requirement of liver transplantation or liver-related death including – among other parameters – CTP score **(model 1)** or UNOS MELD (2016)-score and dACLD **(model 2)** with etiological cure and non-liver-related death as competing risks.

Abbreviations: aSHR – adjusted subdistribution hazard ratio; CTP – Child-Turcotte-Pugh score; dACLD – decompensated advanced chronic liver disease; HVPG – hepatic venous pressure gradient; UNOS MELD (2016) – United Network for Organ Sharing model for end-stage liver disease (2016) score
